# School Absenteeism Longer Than Two Weeks Is a Red Flag of Somatic Symptom and Related Disorders in Hospitalised Children and Adolescents: A Matched Cohort Study

**DOI:** 10.3390/children11060613

**Published:** 2024-05-21

**Authors:** Karen Console, Giorgio Cozzi, Giada Caiffa, Sara Romano, Giulia Gortani, Andrea Clarici, Egidio Barbi, Elena Magni

**Affiliations:** 1 Department of Medicine, Surgery and Health Sciences, University of Trieste, 34127 Trieste, Italyegidio.barbi@burlo.trieste.it (E.B.); 2 Institute for Maternal and Child Health IRCCS Burlo Garofolo, 34137 Trieste, Italy; 3 Clinical Epidemiology and Public Health Research Unit, Institute for Maternal and Child Health IRCCS Burlo Garofolo, 34137 Trieste, Italy

**Keywords:** absenteeism, somatic symptoms, adolescent, hospitalised, children

## Abstract

Chronic school absenteeism is a common problem in childhood and adolescence, and it is frequently observed in patients with somatic symptom and related disorders (SSRDs). This study aimed to determine whether and to what extent the presence of school absenteeism may be a risk factor for the diagnosis of SSRDs in hospitalised patients. This matched cohort study included children and adolescents aged between 8 and 17 years, admitted to the paediatric ward of the IRCCS Burlo Garofolo in Trieste from 2021 to 2023, who were divided into two groups, the first including children with at least 15 days of absence from school for medical reasons and the second including children with regular school attendance, matched to the former group by age and sex. We consecutively enrolled 70 patients, 35 in the absentee group and 35 in the control group. In the absentee group, 30/35 (85.7%) patients were diagnosed with an SSRD, while in the control group, 1/35 (2.9%) was diagnosed with an SSRD. The absentee group had a 30-fold higher risk of being diagnosed with SSRDs than the control group (RR = 30 [95% CI = 4.3–208]; *p* < 0.001). This study shows that in hospitalised children, a history of school absenteeism of more than two weeks is an important risk factor for the diagnosis of SSRDs.

## 1. Introduction

Chronic school absenteeism is a relatively common problem in childhood and adolescence. It is defined as missing school for 10% (or about 18 days) of the academic year [1]. Most children frequently stay home with parental awareness and motivate the absence with medical, familial, or social issues [2]. Chronic school absenteeism may be due to school-related problems, particularly bullying or school difficulties [3]; however, it should also evoke suspicion of somatic symptom and related disorders (SSRDs) when associated with medical reasons.

SSRDs are conditions in which the patient’s subjective reporting of physical symptoms is associated with distress, the disruption of daily functioning, and disproportionate thoughts, feelings and behaviours related to such symptoms [4,5]. SSRDs are highly prevalent in the paediatric population. In the United States, this type of disorder is estimated to contribute 15–20% of the annual paediatric health care cost burden, requiring numerous investigations and hospital admissions. Although prevalence data are limited, estimates range from 17% to 50% of school-age children and adolescents, and potentially 20% of children between the ages of 3 and 5 have experienced or have SSRDs [6]. The recent pandemic also affected the occurrence of this type of disorder among adolescents, and its psychological impact should not be underestimated. An Italian retrospective study showed that there was a relative increase in the rate of admissions for somatic symptom disorders in the year of the pandemic compared with the previous year [7]. Symptoms are usually physical, not relieved by standard medications, and last for months or years. For this reason, affected children experience substantial impairment in daily life and social functioning, and they are usually unable to attend school regularly. In this case, a rehabilitative approach is recommended, emphasizing returning to school as part of returning to normal social function. A multidisciplinary approach to communication and collaboration with the family, school staff, and health professionals is needed [2,8,9,10]. Chronic school absenteeism is not explicitly cited among the diagnostic criteria for SSRDs, even though previous studies have revealed that it is a typical feature of these clinical conditions [11].

This study investigated whether and to what extent chronic school absenteeism may be a risk factor for diagnosing SSRDs in hospitalised children and adolescents. In the second instance, we aimed to evaluate the social and clinical factors most commonly present in patients with a history of chronic school absenteeism and their association with the final diagnosis of SSRDs.

## 2. Materials and Methods

This matched cohort study included patients admitted to the paediatric ward of the tertiary level university teaching children’s hospital, Institute for Maternal and Child Health IRCCS Burlo Garofolo of Trieste, Italy. This Institute is in the northeast of Italy, with a catchment area of about 300,000 inhabitants. The paediatric ward admits about 600 patients per year, mainly from the paediatric emergency department but also patients with complex or chronic illnesses or special diagnostic needs requiring hospitalization, including those from other regions.

The Institutional Review Board of the Institute for Maternal and Child Health IRCCS Burlo Garofolo of Trieste (RC 34/18) approved the study protocol.

Two groups of hospitalised children and adolescents aged 8 to 17 were enrolled between May 2021 and May 2023.

The families of all registered patients consented to the study and the anonymised processing of personal data.

In this study, chronic school absenteeism was defined as absence from school for at least 15 days for medical reasons, which accounts for about 10% of the school year in Italy. Absences, including continuous and intermittent ones, were recorded cumulatively from the beginning of the school year during which hospitalization occurred. In the analysis of absentee patients, we did not consider the “truants”, i.e., children who hide school absences from their parents [12].

The absenteeism group included all hospitalised children and adolescents whose medical history was remarkable for chronic school absenteeism, defined as absence from school for at least 15 days for medical reasons, during the study period in the current school year.

The control subjects were a series of hospitalised patients, with average school attendance (less than 15 days of absence from school for medical reasons since the beginning of the school year). They matched the absentee patients by age and sex in a 1:1 ratio. Patients with critical or acute conditions with presentation unequivocally related to organic causes were excluded from the control group.

Patients with symptoms suggestive of neuropsychiatric disorders at admission and patients with a known intellectual disability were excluded from the study. In patients with symptoms suggestive of neuropsychiatric pathology at admission, a neuropsychiatric consultation was required to assess our diagnostic suspicion.

For all patients, the diagnosis at discharge, made by a paediatrician on the ward who was unaware of the aim of the study, was recorded. However, if the symptoms presented by the patient were suggestive of SSRDs during hospitalisation, psychiatric counselling was requested before discharge to rule out co-morbidities and to refer patients to psychological care.

The diagnosis of SSRDs was made according to the diagnostic criteria of the fifth edition of the Diagnostic and Statistical Manual of Mental Disorders (DSM-V-TR) [4].

Data on patients’ medical history and hospitalization were acquired by administering a questionnaire to patients and their parents and through the consultation of medical records. The questionnaire was specifically developed for the study (Supplementary Table S1).

A 3-month follow-up based on a phone interview was planned for every enrolled patient asking about the child’s clinical condition and whether school absenteeism persisted.

The primary study outcome was to evaluate the relative risk (RR) of chronic school absenteeism in the medical history and a final diagnosis of SSRDs.

The secondary outcomes were:

- To assess the differences between the two groups regarding the following items: social factors (school environment, group of schoolmates, and sports activity), hospitalization course (investigations performed, duration of hospitalization, and diagnoses at discharge), therapies administered (pre- and post-hospitalization), and follow-up.
- To assess the relative risk of selected social and clinical factors (i.e., those that differed significantly between the two groups in the previous analysis) in receiving a diagnosis of SSRDs, considering the entire study population.

### Statistical Analysis

Data were written as numbers and percentages for categorical variables, and differences between the two groups were assessed using the chi-square test or Fisher’s exact test when appropriate. After evaluating their distribution via the Shapiro–Wilk test, continuous variables were presented as the median and interquartile range (IQR). The Mann–Whitney non-parametric test was therefore employed for group comparison. The differences were considered statistically significant for *p*-values below 0.05. The relative risk and its 95% confidence interval (CI) were calculated for the primary and secondary outcomes. All statistical analysis was performed with STATA Statistical Software, Version 18 (2023. StataCorp LLC, College Station, TX, USA).

## 3. Results

### 3.1. Patient Characteristics

From May 2021 to May 2023, 37 consecutive patients met the inclusion criteria for the absenteeism group. Two families refused to consent to the study. Since a 1:1 ratio was established between the two groups, 35 patients were enrolled in the control group. A total of 70 patients were then included. A comparison of baseline characteristics in the absenteeism and control groups is presented in Table 1. The difference between the two groups was statistically significant in the number of school days missed for medical reasons (*p* < 0.001). Regarding the main presenting symptoms, osteo–artro–muscular pain occurred more frequently in the absenteeism group than in the control group (*p* = 0.02), as did thoracic–abdominal pain (*p* = 0.04). Six (17.1%) patients in the absenteeism group used walking aids, and no patients in the control group (*p* = 0.03) used them.

### 3.2. Primary Endpoint

The diagnoses at discharge in the two groups are summarised in Table 2. In the absenteeism group, 30/35 (85.7%) patients were diagnosed with SSRDs, and in the control group, 1/35 (2.9%) was diagnosed with an SSRD; the remaining diagnoses were organic diseases or were not categorised (*p* < 0.001). Thus, the absenteeism group had 30 times the risk of an SSRD diagnosis compared with the control group (RR = 30 [95% CI 4.3–208]; *p* < 0.001) (Table 2).

### 3.3. Secondary Endpoint

#### 3.3.1. Social Factors

Data on school environment and sports activity were collected in both groups (Table 3). The differences between the two groups were significant in the negatively perceived classroom environment (*p* < 0.001), the lack of a relationship with peers (*p* < 0.001), and the discontinuation of sports activities (*p* < 0.001).

#### 3.3.2. Hospitalization

Data on hospitalization are shown in Table 4. The diversity in the seasonal distribution of patients appeared to be significant (*p* = 0.01), with a spike in admissions in the spring period for the absenteeism group.

Merging the pre-admission diagnoses with those made during hospitalization, we revealed that neuropsychiatric comorbidities were present in 13/35 (37.1%) patients in the absenteeism group and 5/35 (14.3%) patients in the control group, showing a statistically significant difference (*p* = 0.03) (Table 4).

#### 3.3.3. Follow-Up and Therapies

A lack of improvement at follow-up occurred in 14/35 (40%) patients in the absenteeism group and in only 4/35 (11.4%) patients in the control group (*p* = 0.01). Collecting data on therapies administered before and after admission shows that 42.9% of absentee patients achieved a reduction in drug therapies received on admission compared with 14.3% of controls. In addition, 60% of absentee patients were referred to a psychotherapy service compared with 8.6% of the children who attended school regularly (Table 4).

#### 3.3.4. Analysis of the Relative Risk of Selected Social and Clinical Factors with SSRD Diagnosis

The relative risk associated with selected clinical (main presenting symptoms, neuropsychiatric comorbidities, and use of aids), social factors (school environment, group of peers, and sports activity), improvement of symptoms, and final diagnoses of SSRDs were assessed. All factors, except for the presence of osteo–articular–muscular pain, significantly increased the risk of receiving a diagnosis of SSRDs, especially the discontinuation of sport activity (RR 12.1, 95% CI 3.1–46.4), as shown in Table 5.

## 4. Discussion

In this study, 85.7% of patients with a history of chronic school absenteeism were diagnosed with SSRDs compared with 2.9% in controls. Moreover, only 5.7% of patients in the absenteeism group were diagnosed with an organic disease. These data confirm that school absenteeism is a decisive risk factor for the diagnosis of SSRDs, with a relative risk of 30 times in hospitalised children and adolescents. Our data are aligned with a more extensive retrospective study involving 243 patients aged 5–18 years diagnosed with SSRDs admitted to a US children’s hospital between 2012 and 2014. Only 10% of these patients attended school regularly the previous year, and more than a third of them had severe school absenteeism [13].

The population we enrolled in was homogeneous regarding the demographic characteristics of the two groups. Concerning symptoms on admission, fever, asthenia, and headache were the most frequent in both groups. Absentee patients reported more abdominal, thoracic, or osteo–artro–muscular pain; these data were then correlated with the diagnosis of SSRDs, and we found that the presence of these symptoms on hospital admission significantly increased the risk of receiving this type of diagnosis (Table 5). Moreover, in our study, such symptoms resulted in the use of walking aids in 17.1% of absentee patients. It is well known in literature that these patients may develop an actual disability due to incongruous medicalization or the inappropriate use of medical aids such as a wheelchair or crutches [14].

Even though 71.4% of the absenteeism group had undergone more than three specialist examinations before admission, 68.6% had more than three laboratory tests, and 97.1% had performed at least one imaging examination, as many as 31.4% had not received a hypothetical diagnosis. Only 5.7% of SSRDs had been previously suspected, emphasizing that, when necessary, paediatricians should make an SSRD diagnosis positive, explaining to the family that it is not a diagnosis of exclusion but that the child is suffering from a definite disease with known and universally recognised diagnostic criteria. The aim is to shift the family’s focus from the continued search for the cause of the symptom to the resumption of the child’s function and sociality, limiting doctor shopping and the risk of a Munchausen by physicians as much as possible [15].

The analysed social factors showed significant differences between the two groups, and, in the secondary analysis, a hostile school environment, the absence of a peer group, and the discontinuation of sports activity resulted in an increased risk of developing SSRDs. This suggests the importance of investigating the social aspects of a patient’s life while taking their history, particularly if SSRDs are suspected. A Swedish study explored the importance of social relationships with parents and peers during adolescence in determining the presence of somatic and functional symptoms in adulthood. The results proved that these symptoms are strongly associated with poor contact with parents and dissatisfaction with classmates at age 16 [16]. The TRAILS study revealed a strong correlation between a low level of physical activity in a cohort of adolescents with SSRDs, a sedentary life, and the onset of the disorder [17].

Hospitalizations of patients in the absenteeism group were more concentrated in the spring months (62.9%), during which there was more significant school pressure. Numerous studies have emphasised that the presenting symptoms of SSRDs follow a characteristic temporal distribution, e.g., they increase in the morning before school, on exam days, or on days following weekends or vacations [12].

At discharge, 42.9% in the absenteeism group achieved a reduction in drug therapies (mainly analgesics), and 48.6% were referred to a psychotherapy service. Psychological therapies (cognitive behavioural therapy, hypnosis, biofeedback, and relaxation) have been proven effective in reducing symptom burden, disability, and absence from school in children with SSRDs [18].

In the absenteeism group, we detected more patients with psychiatric comorbidities compared with controls. In the study population, the presence of neuropsychiatric comorbidities increased the risk of SSRD diagnosis. This finding fits with an Australian study that analysed a cohort of adolescents followed at a neuropsychiatric centre for chronic school absenteeism. It showed that 54% had an anxiety disorder and 52% had depression [19]. A case-control study in Scotland demonstrated that chronic school absenteeism was strongly associated with psychiatric disorders (45% in cases vs. 17% in controls, *p* < 0.001) [20].

In patients diagnosed with SSRDs, the risk of symptom persistence was increased (Table 5). Somatic symptom disorders are generally chronic, with fluctuating symptoms. Studies have shown that patients can recover, with symptoms improving in about 50–75% of patients while getting worse in 10–30% of patients [21,22]. The best prognostic markers include fewer physical symptoms and better functioning at baseline [22,23].

Therefore, the results of our study revealed some prominent features that may help paediatricians suspect SSRDs, including children and adolescents with recurrent abdominal, thoracic, or osteo–articular–muscular pain; who no longer attend their peer group and have quit their usual sports activities; and, most importantly, who have missed many days of school. While our cohort was too limited to draw any firm conclusions, the presence of a very high BMI is also a possible risk factor for school absenteeism and the development of SSRDs and, if confirmed by other studies, could be considered a red flag from a pragmatic perspective. A meta-analysis demonstrated that the risk of being chronically absent from school was 27% and 54% higher in overweight and obese children than in their normal-weight peers [24]. The resumption of school attendance is critical in treating somatic symptom disorders, not only because it leads to the clinical improvement of these patients but also because it safeguards their future, as school attendance problems have also been linked to possible adverse outcomes in adult life [12]. A positive relationship between doctor and patient is also essential. It should be accompanied by frequent and supportive visits, avoiding the temptation to prescribe drugs or tests when these interventions are not clearly indicated [21].

### Strengths and Limitations of the Study

This study’s primary limitations were the limited number of patients enrolled and its monocentric nature. Additional limitations were the use of an unvalidated questionnaire and the fact that school absences for medical reasons were confirmed not by medical personnel but by the presence of excuse notes written by the children’s parents (as legislated by the current legal system in Italy). Finally, the lack of measurements of anxiety levels was another limitation that should be addressed in further studies. The strengths of our study were the homogeneity of the enrolled population, the assessment of the clinical conditions of all enrolled patients after discharge, and a study population of hospitalised patients only.

## 5. Conclusions

In this matched cohort study, we demonstrated that by carefully investigating social factors and analysing the total days of absence from school, paediatricians can better distinguish between purely organic disorders and SSRDs. In particular, a hospital admission with a history of school absenteeism longer than two weeks is a substantial red flag for the diagnosis of SSRDs.

## Figures and Tables

**Table 1:** Baseline characteristics of 35 absentee patients and 35 matched controls.

	Absenteeism N = 35	Controls N = 35	*p*-Value
**Sex male, n (%)**	17 (48.6)	17 (48.6)	1.00
**Age in years, median (IQR)**	13 (11–15)	13 (11–15)	1.00
**Days of absence, median (IQR)**	25 (15–40)	0 (0–3)	**<0.001**
**BMI, median (IQR)**	21.4 (18.2–25)	19.1 (16.4–22.2)	**0.04**
**Medical Comorbidities, n (%)**			0.20
No	26 (74.3)	21 (60.0)	
Yes	9 (25.7)	14 (40.0)	
**Neuropsychiatric comorbidities, n (%)**			**0.03**
No	22 (62.9)	30 (85.7)	
Yes	13 (37.1)	5 (14.3)	
**Pre-hospitalization**			
Previous hospitalizations, n (%)			1
0	24 (68.6)	23 (65.7)	
1	10 (28.6)	10 (28.6)	
2	1 (2.8)	2 (5.7)	
Specialist visits, n (%)			0.06
1	7 (20.0)	14 (40.0)	
2	3 (8.6)	6 (17.1)	
≥3	25 (71.4)	15 (42.9)	
Imaging tests, n (%)			0.24
0	1 (2.9)	6 (17.1)	
1	11 (31.4)	8 (22.9)	
2	9 (25.7)	7 (20.0)	
≥3	14 (40.0)	14 (40.0)	
Laboratory examinations, n (%)			0.64
0	2 (5.7)	1 (2.9)	
1	5 (14.3)	9 (25.7)	
2	4 (11.4)	4 (11.4)	
≥3	24 (68.6)	21 (60.0)	
Previous diagnoses, n (%)			**0.047**
Organic disease	10 (28.6)	20 (57.1)	
Depression/Anxiety	2 (5.7)	0	
SSRDs	2 (5.7)	0	
Other	5 (14.3)	5 (14.3)	
Multiple diagnoses	5 (14.3)	6 (17.1)	
No diagnosis	11 (31.4)	4 (11.4)	
**Clinical symptoms at admission**			
Fever/Asthenia/headache	14 (40.0)	13 (37.1)	1.00
Dyspnoea	2 (5.7)	1 (2.9)	1.00
Osteo–artro–muscular pain	12 (34.3)	3 (8.6)	**0.02**
Thoracic/abdominal pain	12 (34.3)	4 (11.4)	**0.04**
Lumbosacral/cervical pain	1 (2.9)	3 (8.6)	0.61
Weight loss/increase	1 (2.9)	3 (8.6)	0.61
Polyuria/Polydipsia/Vomiting/Diarrhoea	3 (8.6)	5 (14.3)	0.71
**Use of walking aids**			**0.03**
No	29 (82.7)	35 (100)	
Yes	6 (17.1)	0 (0.0)	

IQR: interquartile range.

**Table 2:** Primary endpoint.

Diagnosis	Absenteeism N = 35	Controls N = 35		*p*-Value
**Type of diagnosis at discharge, n (%)**				**<0.001**
Organic nature	2 (5.7)	33 (94.2)		
SSRDs	30 (85.7)	1 (2.9)		
Uncategorizable	3 (8.6)	1 (2.9)		
**Outcome**	**Absenteeism** **n/N**	**Controls** **n/N**	**Risk Ratio [95% CI]**	***p*** **-value**
SSRD Diagnosis	30/35	1/35	30 [4.3; 208]	<0.001

**Table 3:** Secondary endpoints: social factors.

SOCIAL FACTORS	Absenteeism N = 35	Controls N = 35	*p*-Value
**School years lost**, n (%)			0.49
No	33 (94.3)	35 (100)	
Yes	2 (5.7)	0 (0.0)	
**School**, n(%)			0.74
First grade	6 (17.1)	5 (14.3)	
Middle school	17 (48.6)	17 (48.6)	
Professional Institute	2 (5.7)	0 (0.0)	
Technical Institute	4 (11.4)	4 (11.4)	
Gymnasium	6 (17.1)	9 (25.7)	
**School environment**, n (%)			**<0.001**
Negative	18 (51.4)	3 (8.6)	
Positive	17 (48.6)	32 (91.4)	
**Group of schoolmates**, n (%)			**<0.001**
No	15 (42.9)	2 (5.7)	
Yes	20 (57.1)	33 (94.3)	
**Sports activity**, n (%)			0.11
No	13 (37.1)	7 (20.0)	
Yes	22 (62.9)	28 (80.0)	
**Pressure in sports** #, n (%)			0.58
No	20 (90.9)	27 (96.4)	
Yes	2 (9.1)	1 (3.6)	
**Sports interruption** #, n (%)			**<0.001**
No	3 (13.6)	25 (89.3)	
Yes	19 (86.4)	3 (10.7)	

# Considering only those who practised a sport.

**Table 4:** Secondary endpoints (2): hospitalization, follow-up and therapies.

HOSPITALIZATION	Absenteeism N = 35	Controls N = 35	*p*-Value
**Specialist visits**, n (%)			**0.02**
0	3 (8.6)	12 (34.3)	
1	14 (40.0)	5 (14.3)	
2	11 (31.4)	9 (25.7)	
≥3	7 (20.0)	9 (25.7)	
**Imaging examinations**, n (%)			0.07
0	4 (11.4)	10 (28.6)	
1	14 (40.0)	10 (28.6)	
2	9 (25.7)	3 (8.5)	
≥3	8 (22.9)	12 (34.3)	
**Laboratory examinations**, n (%)			0.05
0	7 (20.0)	2 (5.7)	
1	11 (31.4)	6 (17.1)	
2	8 (22.9)	8 (22.9)	
≥3	9 (25.7)	19 (54.3)	
**Period of hospitalization**, n (%)			**0.01**
December–February	11 (31.4)	17 (48.6)	
March–May	22 (62.9)	9 (25.7)	
June–Augusy	0	3 (8.6)	
September–November	2 (5.7)	6 (17.1)	
**Duration of hospitalization**, days med (IQR)	4 (3–5)	6 (3–8)	0.05
**Neuropsychiatric comorbidities diagnosed during hospitalization**	7 (20)	2 (5.7)	**0.15**
Neuropsychiatric comorbidities diagnosed pre and during hospitalization	13 (37.1)	5 (14.3)	**0.03**
**FOLLOW-UP**, n (%)			**0.01**
Improvement of symptoms	21 (60.0)	31 (88.6)	
Persistence of symptoms	14 (40.0)	4 (11.4)	
**THERAPIES**			
**Neuropsychiatric therapy**, n (%)			**<0.001**
No	14 (40.0)	32 (91.4)	
Psychotherapy	17 (48.6)	3 (8.6)	
Psychotherapy and medication	4 (11.4)	0 (0.0)	
**Comparison drug therapy pre/post hospitalization**, n (%)			**0.02**
Stable	14 (40.0)	16 (45.7)	
Reduction in the number of drugs prescribed	15 (42.9)	5 (14.3)	
Increase in the number of drugs prescribed	6 (17.1)	14 (40.0)	

**Table 5:** Relative risk of selected characteristics to receive a final diagnosis of SSRDs.

	SSRD Diagnosis	Risk Ratio [95% CI]	*p*-Value
**Social factors**			
School environment		2.2 [1.3; 3.6]	**0.003**
Negative, n/N	15/21		
Positive, n/N	16/49		
Group of schoolmates		2.6 [1.6; 4.0]	**<0.001**
No, n/N	14/17		
Yes, n/N	17/53		
Sport interruption #		12.1 [3.1; 46.4]	**<0.001**
Yes, n/N	19/22		
No, n/N	2/28		
**Clinical factors**			
Thoracic/abdominal pain		2.1 [1.3; 3.4]	**0.005**
Yes, n/N	12/16		
No, n/N	19/54		
**Osteo–artro–muscular pain**		1.5 [0.9; 2.5]	0.17
Yes, n/N	9/15		
No, n/N	22/55		
**Neuropsychiatric comorbidities**		1.8 [1.1; 3]	**0.03**
Yes, n/N	12/18		
No, n/N	19/52		
**Use of mobility aids**		2.5 [1.8-3.3]	**0.01**
Yes, n/N	6/6		
No, n/N	25/64		
**Improvement of symptoms**		1.9 [1.3; 2.9]	**0.006**
No, n/N	14/18		
Yes, n/N	21/52		

# Considering only those who practised a sport.

## Data Availability

All relevant data are included in the article and/or its supplementary information files. All other data supporting the study are available from the corresponding author upon request. The data are not publicly available due to privacy reasons.

## Supplementary Materials

The following supporting information can be downloaded at: https://www.mdpi.com/article/10.3390/children11060613/s1, Table S1: Data collected in patient enrolment (questionnaire); Table S2: Detailed comorbidities.
