# Supplementary material for: School Absenteeism Longer Than Two Weeks Is a Red Flag of Somatic Symptom and Related Disorders in Hospitalised Children and Adolescents: A Matched Cohort Study
Source: Children (Basel). 2024 May 21;11(6):613. doi: 10.3390/children11060613 (PMC11202215; doi:10.3390/children11060613)
Supplement: Supplementary file 1 [file children-11-00613-s001.zip › children-3008537-supplementary.pdf]

Supplementary Table

Table S1 - Data collected in patient enrolment (questionnaire)

Age<br>Sex<br>BMI
Medical comorbidities<br>Neuropsychiatric comorbidities<br>Previous laboratory tests*<br>Previous imagine exams*<br>Previous specialist visits*<br>Previous hospitalizations*<br>Previous diagnoses*
School<br>- Days of absence from the beginning of the school year<br>- Type of school<br>- School environment#<br>Group of schoolmates
Sport<br>- Sports activity<br>- Pressure in sports<br>Sports interruption
Main symptoms at admission<br>Use of walking aids<br>Period of hospitalization<br>Duration of hospitalization<br>Laboratory tests performed during hospitalization<br>Imaging tests performed during hospitalization<br>Specialist visits performed during hospitalization<br>Diagnosis at discharge<br>Drugs at admission<br>Drugs prescribed at discharge<br>Psychiatric therapy prescribed at discharge<br>Follow up

* Concerning the same symptom, they were hospitalized at the Pediatric ward of the IRCCS Burlo Garofolo.

Table S2 – Detailed comorbidities

	Absenteeism<br><br>N=35	Controls<br><br>N=35
Comorbidities (n)	9	14
	2 Hashimoto's thyroiditis<br>2 Obstructive Sleep Apnea Syndrome<br>1 Obesity-associated chronic dermatitis<br>1 Dermatomyositis<br>1 Congenital renal pyelectasis<br>1 G6PDH deficiency associated with presence of cochlear implant<br>1 Polycystic ovarian syndrome	2 Asthma,<br>2 Ulcerative colitis<br>2 Diabetes type 1<br>1 Sclerosing cholangitis<br>1 Celiac disease<br>1 Achondroplasia associated with celiac disease<br>1 Scoliosis<br>1 Epilepsy<br>1 Turner syndrome associated with Hashimoto's thyroiditis<br>1 Blue rubber bleb nevus syndrome<br>1 Cystic fibrosis
Neuropsychiatric comorbidities (n)	13	5
	4 Depressive disorder<br>4 Anxiety disorder<br>2 Anxiety disorder associated with depressive disorder<br>1 Attention-Deficit/Hyperactivity Disorder (ADHD) associated with anxiety disorder<br>1 Social phobia<br>1 Post-traumatic stress disorder	2 Anxiety disorder<br>2 Autism spectrum disorder<br>1 Depressive disorder
